# Teaching and the acquisition of complex and opaque cultural skills and knowledge among the Koygu hunter-gatherers and Nyangatom pastoralists of southern Ethiopia

**DOI:** 10.3389/fpsyg.2026.1849924

**Published:** 2026-10-15

**Authors:** Temechegn G. Bira, Barry S. Hewlett, Edward H. Hagen

**Affiliations:** 1 Department of Anthropology, Washington State University, Pullman, WA, United States; 2 Department of Sociology and Social Anthropology, Arba Minch University, Arba Minch, Ethiopia; 3 Department of Anthropology, Washington State University, Vancouver, WA, United States

**Keywords:** Children, opaque culture, complex skill, teaching, pastoralist, southern Ethiopia

## Abstract

In order to adapt to diverse ecological and social settings, humans learn skills, norms, and knowledge from others. These skills and knowledge range from relatively transparent skills to complex cultural norms and rituals. The study utilized focal-follow behavioral observations of 60 children aged 3–18 years, to evaluate cultural evolutionary and social learning predictions about the relationship between teaching and learning opaque and complex skills and knowledge in small-scale cultures. Each child was observed for 2 hours during daylight hours between 6:00 a.m. to 6:00 p.m. Data were gathered from the Koygu transitional hunter-gatherer and Nyangatom pastoralist cultures in southern Ethiopia. The study found that (1) teaching generally increased with child age, especially among the Nyangatom; (2) Nyangatom children were more likely to learn from their parents and other adults, whereas Koygu children were more likely to learn from other children, including siblings; (3) teaching was significantly related to the opacity of the skill or knowledge, but was not related to skill complexity, and (4) the relative proportion of transparent, complex and opaque skills or knowledge did not vary by child age.

## 1 Introduction

How do children learn cultural knowledge that is too complex or opaque to be acquired through simple observation? In order to adapt to diverse ecological and social settings, humans learn skills, norms, and knowledge from others. Children grow up not merely in physical environments, but within culturally constructed niches filled with artifacts, skills, beliefs, and practices that have been inherited, accumulated, and modified over generations (Legare, 2017). What social learning mechanisms enable humans to acquire particularly complex features of culture with sufficient fidelity to sustain cumulative culture?

Teaching, i.e., modifying one’s behavior to facilitate someone else learning a skill or knowledge (Hewlett et al., 2011; Kline, 2015), has been proposed as a possible solution to this occurring evolutionary problem. However, it is contested by various researchers. On the one hand, some suggest that teaching is rare or does not exist in small-scale societies (Lancy, 2010; Lancy and Grove, 2010). On the other hand, others argue that teaching is a human universal (Csibra and Gergely, 2011; Kline, 2015; Tomasello, 2016) and a significant and relatively unique process in human social learning (Legare, 2017). Cross-cultural studies in small-scale cultures have found that teaching not only exists but is also a major process of social learning (Bira and Hewlett, 2023; Garfield et al., 2016). Field studies especially among hunter-gatherers indicate that teaching is more subtle, takes place in the context of touching and close proximity, and that children learn with minimal overt interventions (Boyette and Hewlett, 2018; Hewlett and Roulette, 2016). Among researchers who support the existence of teaching across cultures, an ongoing research issue concerns the claim that teaching is primarily associated with the transmission of culturally complex or opaque that emerge later in childhood and adolescence (Garfield and Lew-Levy, 2025; Salali et al., 2019). These conflicting perspectives may be understood by examining how, when, and for what kinds of knowledge teaching is actually utilized.

In this paper, we examine the roles of teaching in the acquisition of opaque and complex cultural traits among two culturally distinct societies in southern Ethiopia: Nyangatom agopastoralists and Koygu hunter-gatherers. Following Lyons et al. (2007), we used the term opaque culture to refer to causal opacity which implies to those tasks in which the relationship between an action and its intended goal is not directly perceivable, whereas *complex cultural skills* implies to multi-step instrumental culture that involves a degree of cognitive and social skill requiring a sequence of intricate, multi-step actions (Buskell, 2022) and rely heavily on repetitive learning involving observation, local enhancement, and teaching depending on their processing complexity (Garfield and Lew-Levy, 2025). Rather than asking whether teaching exists, we ask a more specific questions: when is teaching used, and what problem does it solve? By comparing age-related patterns of teaching, who teaches whom, and whether opaque and complex skills are preferentially transmitted through teaching, we investigate how culturally constructed niches shape the selective deployment of teaching as a social learning mechanism.

We adopt a broad definition of teaching noted above because it encompasses not only explicit verbal instruction, but also subtler forms such as teasing, task assignment, storytelling, stimulus enhancement, commands (safety, invitation and general), child asking questioning and other answering, feedback, and demonstration. Within this framework, teaching need not resemble formal schooling. It is embedded in everyday activity and easily overlooked (Hewlett and Roulette, 2016). The apparent subtlety of teaching may therefore reflect its integration into daily life rather than its absence.

Crucially, teaching is not expected to occur uniformly across all domains of skills and knowledge. Human cultural traits are cumulative: they increase in diversity and complexity over time resulting in phenomena that no one individual could invent alone (Boyd and Richerson, 1996; Tomasello et al., 1993). It becomes cumulative and requires particular kinds of social learning mechanisms that allows the accumulation of successful modifications over time through a process of cultural ratcheting (Tennie et al., 2009; Tomasello, 2016). This process depends on high-fidelity transmission. High-fidelity transmission is necessary for cumulative culture as it ensures cultural traits exist in a population long enough to be modified or combined to produce new variants and traits (Lewis and Laland, 2012; Tennie et al., 2009; Tomasello et al., 1993). As cultural traits become more complex, cumulative cultural transmission may require teaching to ensure accurate transmission from one generation to the next (Fogarty et al., 2011). From this perspective, teaching is not simply another process of learning, but an essential feature of cumulative culture.

Age further complicates this picture. Across childhood and adolescence, children acquire a mix of relatively transparent skills (e.g., tool use, food processing) that are easy to observe as well as abstract or opaque skills or knowledge (e.g., social norms, moral expectations, role obligations). Existing research predicts a developmental shift: early learning is expected to rely primarily on observation and imitation, while teaching is expected to become increasingly important later in childhood and adolescence in order to learn abstract knowledge and more complex skills (Garfield and Lew-Levy, 2025; Salali et al., 2019), as cognitive capacities for abstraction and explicit instruction mature (Dumontheil, 2014). It has been argued that teaching provides children with the opportunity to learn information too complex and abstract to discover on their own such as social norms (Garfield and Lew-Levy, 2025; Legare, 2019; Salali et al., 2019). Teaching is therefore often framed as a late-developing solution to acquiring abstract and complex culture.

Empirical findings complicate this developmental narrative. Although teaching is generally expected to increase with age (Garfield and Lew-Levy, 2025), some forms of teaching such as task assignment appear to decline with age (Boyette and Lew-Levy, 2020). In addition, cross-cultural studies of cultural learning in pastoralists found that the majority (more than 50%) of what children learn occurs in early childhood and before age seven (Bira and Hewlett, 2023). Moreover, studies in pastoralist societies suggest that children assume meaningful social and economic responsibilities at relatively young ages (Hames and Draper, 2004; Lancy, 2015; Tian, 2024), implying that complex and abstract cultural traits may be introduced earlier than researchers predict. These findings raise an important question: Is teaching primarily a late developmental process, or does it scaffold complex learning across much of childhood? Is learning complex cultural traits a late childhood developmental process?

Answering these questions requires attention to the culturally constructed niches in which children learn. Foundational schema, social structures, settlement patterns and proxemics are part of these niches to which children have to adapt. In hunter-gatherer societies, their foundational schema—ways of thinking and feeling that influence many domains of life (Shore, 1996)—emphasizes egalitarianism and respect for individual autonomy which allows children to participate in a broad range of activities where adults seldom intervene in children’s activities or provide direct instruction (Boyette and Hewlett, 2017; Hewlett et al., 2011; Lew-Levy et al., 2018). In contrast, societies with more hierarchical foundational schema, such those among small-scale farmers, where children are expected to respect and obey the requests of elders (especially male elders) which can result in more adult-centered instruction (Boyette and Hewlett, 2017). A comparative study of Aka foragers and Ngandu farmers indicated that Ngandu farming children received three times more instruction than Aka forager children (Boyette and Hewlett, 2017), reflecting divergent assumptions about autonomy and adult transmission of culture.

Subsistence systems further shape these dynamics. In pastoralist and agricultural societies, children are often integrated into productive labor early in life and are assigned tasks that require coordination, foresight, and adherence to norms (Hames and Draper, 2004; Lancy, 2015, 2016). Such contexts may both increase the prevalence of abstract and complex cultural knowledge and heighten the need for teaching to ensure accurate transmission. By contrast, children in foraging societies often have an extended period of freedom from social responsibility (Hewlett et al., 2011) that can last up until the time of marriage. Furthermore, the nature of task assignment to children also varies across subsistence systems. Subsistence work can be limited among forager children (see Marlowe, 2010, studies of Hadza children for an exception), and much of children’s work in these societies is self-initiated and self-guided (Barry et al., 1959; Stieglitz et al., 2013), whereas children in agricultural and pastoral societies are more likely to be assigned tasks by adults (Gurven and Kaplan, 2006; Hames and Draper, 2004), reflecting greater adult expectations for early participation in productive activities. Cultural groups also vary in the degree to which children are segregated from or are participating in adult economic and social activity (Gauvain and Munroe, 2009; Keller, 2007), which in turn shape both opportunities for learning and the social organization of teaching itself.

Despite the theoretical importance of these contrasts, pastoralist societies remain underrepresented in empirical research on teaching and cultural transmission. Pastoralism is a delayed-return system of resource management that requires future-oriented planning and often involves hierarchical social relations and age-graded authority (Barry et al., 1959; Bedasso, 2016; Legesse, 1973; Tornay, 1981). These features make pastoralist contexts especially informative for testing cultural evolutionary models linking teaching to the transmission of complex cultural traits. By comparing Nyangatom pastoralists and Koygu hunter-gatherers, this study asks whether teaching is more frequent when cultural knowledge is more opaque or complex and how teaching may vary by age and ethnic group.

### 1.1 Predictions

1. Given their age-based hierarchy, we predicted that Nyangatom children will be more likely to learn from parents and other older individuals, whereas Koygu children will be more likely to learn from peers, due to their egalitarian foundational schema, higher fertility and dense living conditions.
2. Given previous studies that suggest a developmental shift in the process of learning (Garfield and Lew-Levy, 2025; Salali et al., 2019), we predict that teaching will increase with age, with adolescents receiving more teaching than younger children.
3. Given theoretical and ethnographic studies that show that opaque knowledge are acquired later in life (Garfield and Lew-Levy, 2025; Salali et al., 2019), we predict that opaque and complex cultural traits will be disproportionately transmitted later in childhood and adolescence by teaching.

## 2 Materials and methods

The Koygu and the Nyangatom are located in the Nyangatom district in the South Ethiopia Region (see Figure 1) on the west sides of the Omo River (Bedasso, 2016; Bira, 2019; Buffavand, 2018; Gebre, 2016; Lewis and Woodburn, 2007; Turton, 1986). The district is located at the crossroads between Ethiopia, Kenya and South Sudan (Alemu, 2018). However, the two cultural groups differ substantially in culture, language, subsistence (see Bedasso, 2016; Lewis and Woodburn, 2007), foundational schema and demography (Bira, 2026).

**Figure 1:**
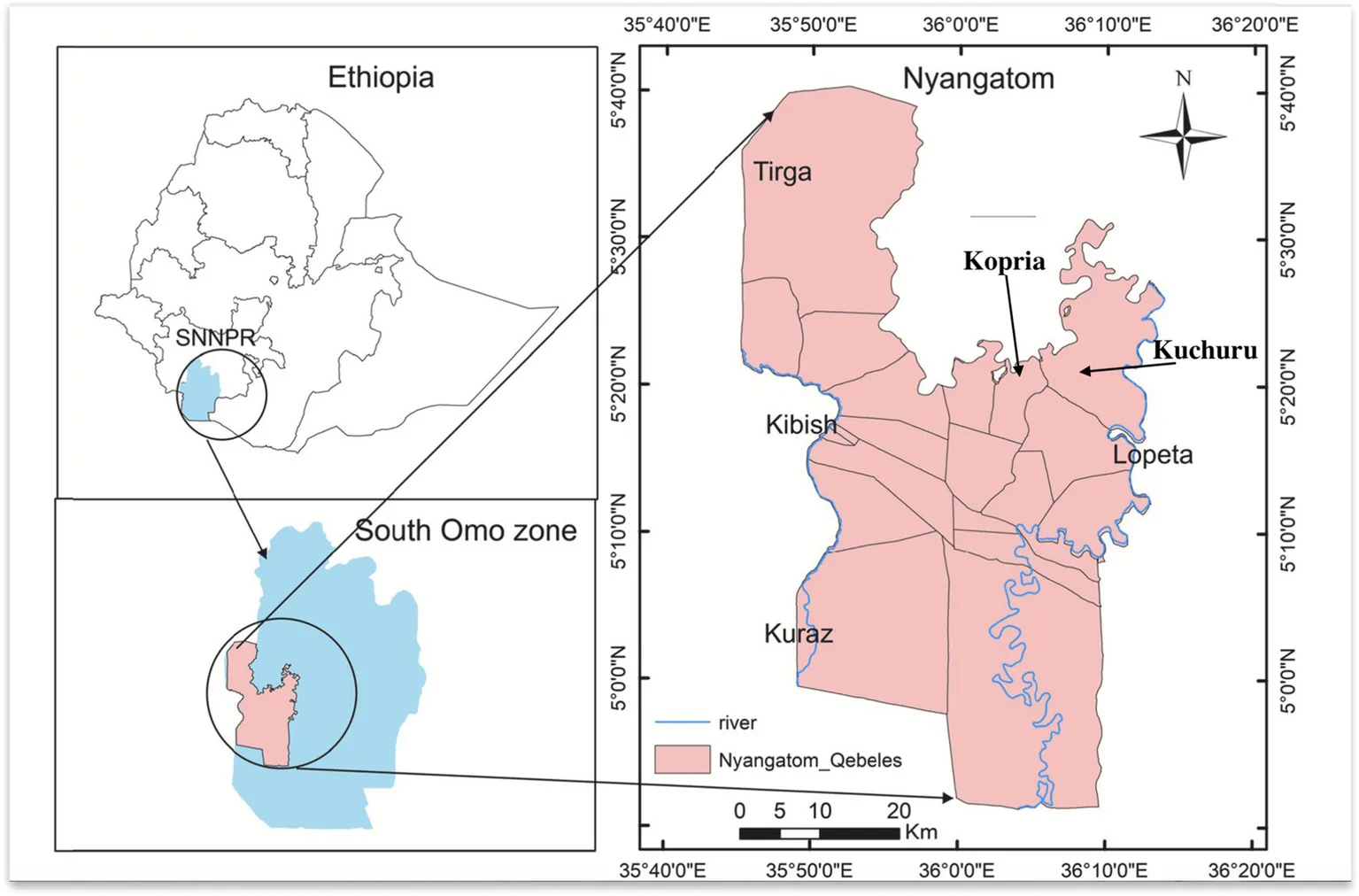
The map of Nyangatom district and the villages (Adapted from Gebeyehu et al., 2021). The fieldwork for the current project was conducted in Kopria and Kuchuru.

### 2.1 Nyangatom agropastoralists

The Nyangatom are more populous than the Koygu with an overall with a population of 25,252 (Central Statistical Agency, 2007). Their territory extends broadly between the Omo River to the east and the Kibish River to the west. They speak the Nyangatom language, which belongs to the Nilotic branch of the Nilo-Saharan language family (Bender, 1976), the same linguistic sub-family spoken by the Turkana and Topossa.

Traditionally, the Nyangatom territory is divided into seven territorial sections (*ekitela*), which includes: Ngilinkakol, Ngisakol, Ngikapung, Ngikor (also called Ngtokoroman), Ngikumama, Ngibunei, and Ngignarich (historically occupied by the Murle) (Bedasso, 2016; Tornay, 1981). Tornay emphasized that territorial section membership is a central marker of Nyangatom identity. As he noted, “Membership to a territorial section is a fundamental social parameter for any Nyangatom. A newcomer or the first time two unfamiliar Nyangatom meet is first identified by inquiring about his section, then his generation and his age-set” (Tornay, 1981, p 159). Territorial section affiliation is patrilineal and remains constant even when individuals relocate. The fieldwork for this research was carried out in the Ngikapung section.

The Nyangatom society is organized into an age-based generational hierarchy with five levels: Ngitome (elephants) being the oldest group; Ngimurkopir (ostriches); Ngingoletiang (ibexes); Ngikosowa (buffaloes) and Ngungoleita (oryx/antelopes) being the youngest group (Bira, 2026). This hierarchy governs daily interactions, seating arrangements, decision-making, and other cultural practices, reflecting a stratified but cohesive society where respect for elders is paramount. Tornay (1981) described the Nyangatom generational system as rooted in the father-son relationship, with sons entering the generation immediately following their fathers. Bedasso (2016) corroborated this, and the first author’s research aligns with this structure. During the time of fieldwork for this study (August 2023–July 2024), all the member of Ngungoleita (the younger generational group) were bachelor and had no children. Their sons will form a new generational group and take on a new name through a naming ceremony called *ekumuomuor.* Nyangatom women are also part of this generation system but enter into it upon marriage taking their husband’s generation (Bira, 2026).

Nyangatom usually identify themselves as pastoralists (Bira, 2026). They keep cattle, goats and donkeys. In addition to this, they also engage in rainfed and flood-recession cultivation (Bedasso, 2016; Bira, 2019; Glowacki and Molleman, 2017; Tornay, 1981).

The Nyangatom settlements are dispersed, and the villages are not densely populated, the dwellings within each village are only a few feet apart, connected by open paths and shared entrances. Each village is fenced with tree branches, only few houses per village and adjacent to the settlement lies an open area that includes grazing land and farmland. In Kopria (see Figure 1), the field site, 9 villages were included in this study. The distance between the villages ranges from about 0.25 to one mile, and the total stretch across the study area is roughly 3.77 miles. Compared to the Koygu, Nyangatom household sizes are relatively small: the mean household size is 4.4 people, and the median is 4.0 (*N* = 100 households). The mean number of live births over a woman’s life (45+) is 5.5 (Bira, 2026).

### 2.2 Koygu transitional hunter-gatherers

The Koygu community live in three isolated villages namely: Chalaka (the most northern village), Kuchuru (the central village and the field site), and Galgida (the most southern village) (Lewis and Woodburn, 2007). Currently all of them live on the western bank of the Omo River (Bira, 2026) and commonly known as a riverine people (Lewis and Woodburn, 2007). These three villages form one administrative unit known as the Kuchuru kebele (see Figure 1). Kebele is the smallest administrative unit in the current administrative structure of Ethiopia. Of the three villages, Kuchuru is the largest of the with a population of about 754 out of a total estimated population of 1,260 (Bira, 2026). It is approximately 45 km from the nearest market town, Kangaten (Teklehaymanot and Giday, 2010), connected only by a seasonal dirt road with no public transport. The only public infrastructure in the area is a primary school (grades 1–8) located in Kuchuru.

The Koygu language is known as Toga Koygu which means Koygu language. It belongs to the Southeast Surmic within the Surmic language of the Nilo-Saharan language families (Hieda, 1991, 1993). The Koygu are multilingual, and everyone interviewed reported speaking at least one additional language besides their own.

Kuchuru village is fenced by a large wooden fence with two gates. They are in large, settled villages because of political insecurity (Bira and Hewlett, 2023) in the area (i.e., ethnic conflicts). Observations indicate that inside this larger fence, smaller fenced compounds house clusters of two to five households, typically extended families including grandparents, married sons and their wives, and children. Houses are made of grass thatch and are built only a few feet apart, with open paths and shared entrances. Next to the village is an open playfield located between the settlement and the school, where children often gather and play.

The surrounding landscape consists of riverine forests, savannah grassland, and farmland along the Omo River. The Koygu practice flood-recession cultivation from September to October and during a good season rainfed cultivation from December to January using digging stick by clearing the land by machete (Buffavand, 2018; Hieda, 1993; Woodhead, 1982). They use the fertile soil and silt provided by the Omo River to cultivate crops such as sorghum, maize, kidney bean (Lewis and Woodburn, 2007). They also grow tobacco in the riverbank (Bira, 2026). Both men and women engage in these activities. Mostly men prepare the land whereas women plant it. Additionally, they produce honey using traditional beehives known as *wari*, placed mostly in the riverine forests on the eastern side of the Omo (Bira, 2026; Matsuda, 2008; Turton, 1986). Hunting (*adma*) and fishing (*qohunen*) are also important subsistence activities that the Koygu engage in (Buffavand, 2018; Lewis and Woodburn, 2007). Although the Koygu engage in cultivation using digging sticks, honey-production, fishing and hunting, they identify themselves and identified by their neighbors as hunters (Bira, 2026; Lewis and Woodburn, 2007). They share a foundational schema common to other hunter-gather cultures; including egalitarianism, respect for individual autonomy and sharing (see Bira, 2026 for details of Koygu foundational schema and Hewlett et al., 2011 for details of hunter-gatherer foundational schema).

Demographic patterns offer further insight into children’s social ecologies. Koygu households are relatively larger. The mean household size is 4.7 people, and the median is 5.0 (*N* = 154 households), slightly higher than that of Nyangatom communities. The mean number of live births over a woman’s life (45+) is 7.3 (Bira, 2026). Together, the physical environment where big village in which houses are few feet apart and the high fertility among the Koygu suggest that children have more siblings and other children to learn from.

### 2.3 Study sample

For the current study, thirty children and adolescents aged 3–18 years-old were selected for behavioral observations from each of the communities, i.e., the Koygu and the Nyangatom. However, the data for one middle-aged Nyangatom male child was discarded in consultation with the parents because, in the middle of the observation, we were informed by the parents that the child had health issues. We adopted age categories used in developmental psychology: early childhood (3–6 years), middle childhood (7–12 years), and adolescence (13–18 years). Our sampling aimed for equal representation across age and gender. Each child was followed and observed twice, once in the morning and once in the afternoon, for a total of 2 hours each during daylight hours (6 a.m. to 6 p.m.). A focal child was initially identified, and permission was obtained from the child’s parents 2 weeks before starting the observation. Once a child was selected, the parents were contacted 1 day in advance to confirm their availability for the following day and to ask where they and their child would be in the morning. If they agreed, they were informed that the researcher would arrive at a randomly selected time between 6:00 a.m. and 12:00 p.m. at the place that they mentioned. Thus, focal children were selected in consultation with their parents, while observation times were randomly allocated. To capture learning activities throughout the day, the observation time was distributed across the entire day (from 6:00 a.m. to 6:00 p.m.) with some hours having more observations than others. Most of the observations were conducted on the same day with few exceptions.

### 2.4 Analysis

The data set includes a total of 341 learning events (143 among the Koygu and 198 among the Nyangatom) and 465 activities (188 among the Koygu, and 277 among the Nyangatom) collected over 120 h of observation. A learning event refers to a distinct instance in which skills or knowledge are transmitted to a focal child. An activity, on the other hand, denotes individual acts of transmission that occur within a learning event. A single learning event may include multiple activities such as demonstrations, verbal instructions, or collaborative tasks each contributing to the overall transmission process. A total of 39 different skills and knowledge were recorded through focal observation.

The data were coded and analyzed using R to understand patterns of social learning among Koygu and Nyangatom children and to evaluate evolutionary and ethnographic hypotheses about social learning. The results were used to test the theoretical and ethnographic predictions about social learning. Chi-square tests and Generalized Linear Mixed Models (Perktold et al., 2024; McCulloch, 1996; McHugh, 2013) were used to evaluate if the results were statistically significant. All Generalized Linear Mixed Models included participant ID and/or village ID as random intercepts to account for repeated measures and clustering of learning events within individuals and/or within villages. For models with an individual random effect, a village random effect was only included if it improved model fit by AIC.

## 3 Results

### 3.1 Prediction 1

Given their age-based hierarchy, we predicted that in Nyangatom, children will be more likely to learn from parents and other older individuals, whereas Koygu children will be more likely to learn from peers, due to their egalitarian foundational schema, higher fertility and dense living conditions.

In order to examine prediction 1, we utilized the three modes of transmission mathematically modeled by Cavalli-Sforza and Feldman (1981), namely vertical (parents-to-child), horizontal (from peers) and oblique (from non-parental adults to child). See Figure 2.

**Figure 2:**
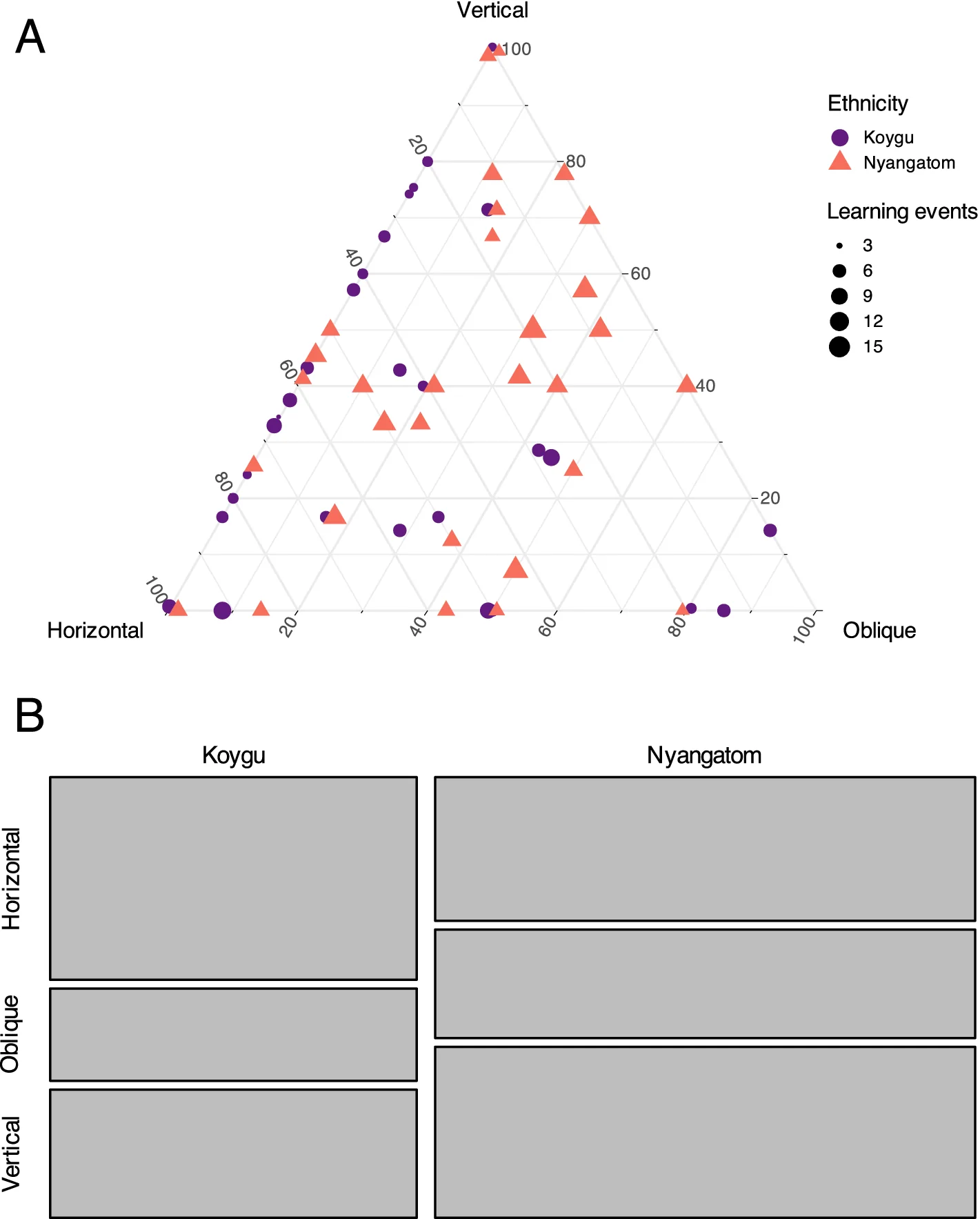
Comparison of Nyangatom and Koygu modes of transmission. **(A)** Ternary plot of transmission modes. Each symbol is one child. Positions on the plot indicate the proportions of horizontal, vertical, and oblique transmission events for that child in the two-hour observation period. Symbol size represents the total number of transmission events in that time period. **(B)** Proportions of each transmission mode among all transmission events for each ethnic group.

To test Prediction 1, we fit separate generalized linear mixed models with a Poisson distribution for the numbers of horizontal, vertical, and oblique transmission events observed during a two-hour period for each child by age category, ethnic group, and their interaction, with a random intercept for village. Consistent with Prediction 1, Nyangatom children were more likely to learn from parents (*p* < 0.05) and non-parental adults (*p* < 0.01) than Koygu children, whereas Koygu children were more likely to learn horizontally from other children including their siblings (*p* < 0.05). See Table 1 and Figures 2, 3.

**Table 1:** Coefficients of generalized linear mixed models of transmission modes, teaching, and opaque/complex vs. transparent learning, by age, ethnicity, and their interaction, with a random intercept for village or individual.

Items	Horizontal	Vertical	Oblique	Teaching	Opaque	Complex
(Intercept)	1.084^***^	0.610^*^	0.269	−0.131	−2.276^***^	−0.367
	(0.110)	(0.245)	(0.170)	(0.169)	(0.344)	(0.294)
Age. *L*	0.000	0.186	−0.668^*^	0.166	−0.192	−0.358
	(0.209)	(0.210)	(0.315)	(0.285)	(0.319)	(0.333)
Age. *Q*	−0.616^***^	0.509^+^	−0.375	0.857^**^	−0.051	−0.403
	(0.172)	(0.274)	(0.273)	(0.297)	(0.336)	(0.338)
Ethnicity-Nyangatom	0.042	0.639^*^	0.578^**^	0.138	1.072^**^	0.943^*^
	(0.152)	(0.276)	(0.209)	(0.229)	(0.373)	(0.386)
Age. *L* × Ethnicity-Nyangatom	−0.306	0.362	0.967^*^	0.954^*^		
	(0.279)	(0.297)	(0.378)	(0.389)		
Age. *Q* × Ethnicity-Nyangatom	0.532^*^	−0.295	0.392	0.004		
	(0.248)	(0.328)	(0.344)	(0.404)		
Process type-Teaching					1.232^***^	−0.066
					(0.296)	(0.219)
SD (Intercept ID)				0.538	1.126	
SD (Intercept Village)	0.000	0.200	0.000			
Num-Obs.	59	59	59	652	652	652

^+^*p* < 0.1; ^*^p < 0.05; ^**^p < 0.01; ^***^p < 0.001; Numbers in parentheses are standard errors. The Horizontal, Vertical, and Oblique models are Poisson with a log link. The Teaching model is binomial with a logit link. The Opaque and Complex coefficients are from a multinomial logistic regression model of skill transmission, with Transparent as the base level.

**Figure 3:**
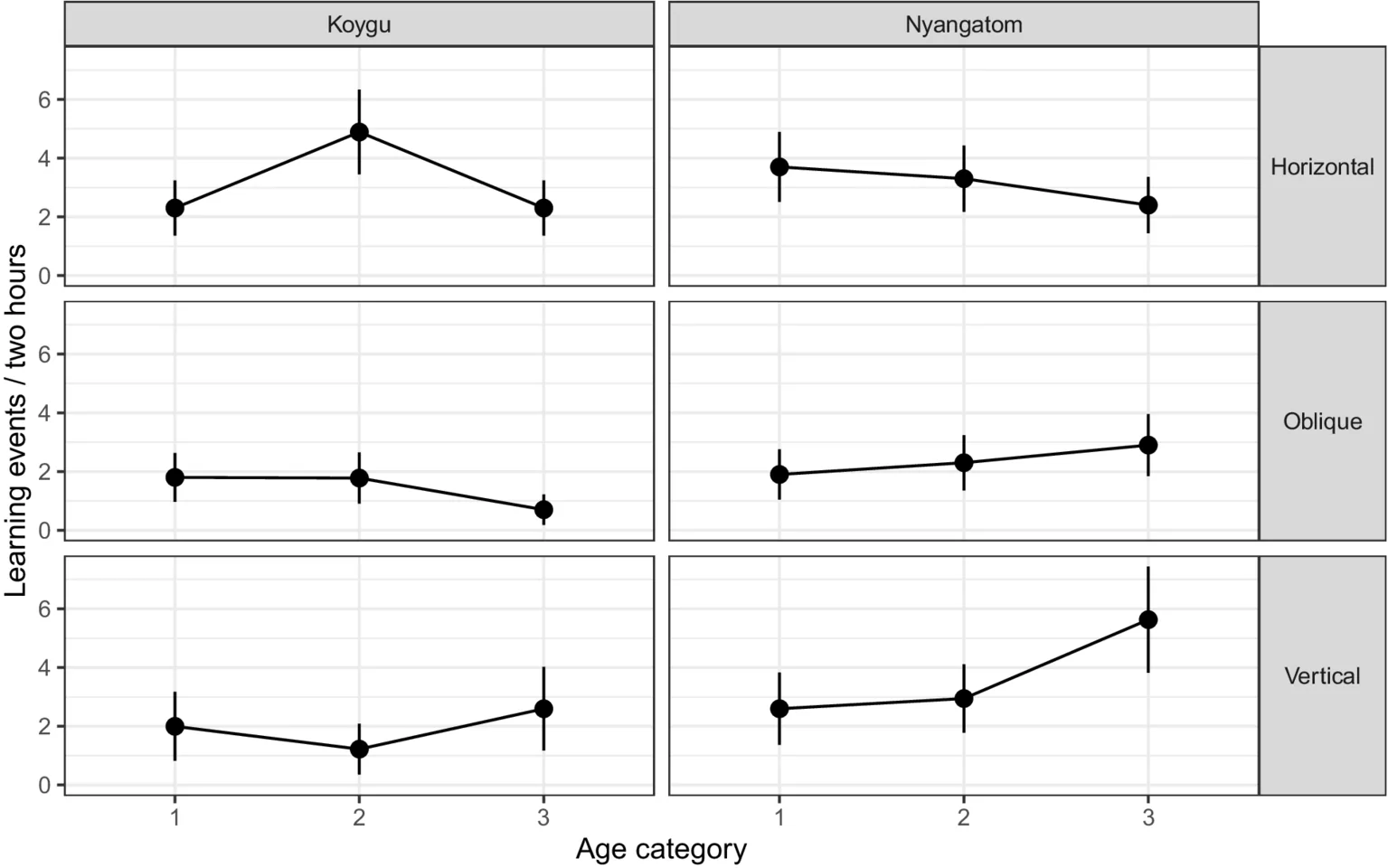
Effects plots of the generalized linear mixed effects models of transmission modes by age category, ethnicity, and their interaction, with a random intercept for village. Models were fit with a Poisson distribution and log link. Error bars are 95% confidence intervals. Age category 1: Early childhood (3–6 years); 2: Middle childhood (7–12 years) and 3: Adolescence (13–18 years). For regression coefficients, see Table 1.

The variability along the modes of transmission across the two groups becomes more evident when examined across specific age categories. For horizontal transmission, there is a statistically significant quadratic age term (*p* < 0.001), as well as an interaction between ethnicity and the quadratic age term, such that horizontal transmission increases in middle-aged Koygu children relative to other children (*p* < 0.05). For oblique transmission, there is a statistically significant linear age term (*p* < 0.05), as well as a significant interaction between age and ethnicity (*p* < 0.05), such that oblique transmission decreases in older Koygu children but increases in older Nyangatom children. See Table 1 and Figure 3.

### 3.2 Prediction 2

Given previous studies that suggest a developmental shift in the process of learning (Salali et al., 2019; Garfield and Lew-Levy, 2025), we predict that teaching will increase with age, with adolescents receiving more teaching than younger children.

The results were mixed. A generalized linear mixed model was used to examine how the likelihood of teaching as a process of learning varies by child age, ethnic group, and their interaction. The main effect of ethnicity was not statistically significant (*β* = 0.14, *p* > 0.05), indicating no overall difference in the probability of teaching between Nyangatom and Koygu at the mean child age. However, the significant age-by-ethnicity interaction indicates that teaching becomes more likely among Nyangatom relative to Koygu as children grow older (linear interaction: *β* = 0.95, *p* = 0.014).

For Koygu, the linear effect of age was not statistically significant (*β* = 0.16, *p* = 0.56), indicating no simple monotonic increase in teaching with age. In contrast, the quadratic age effect was positive and statistically significant (*β* = 0.86, *p* = 0.003), indicating a non-linear relationship between age and teaching. Teaching is more prominent at particular stages of childhood rather than increasing steadily with age. This pattern is illustrated in Figure 4, where teaching frequency is relatively high in early childhood (52%), declines in middle childhood (31%), and increases again during adolescence (55%).

**Figure 4:**
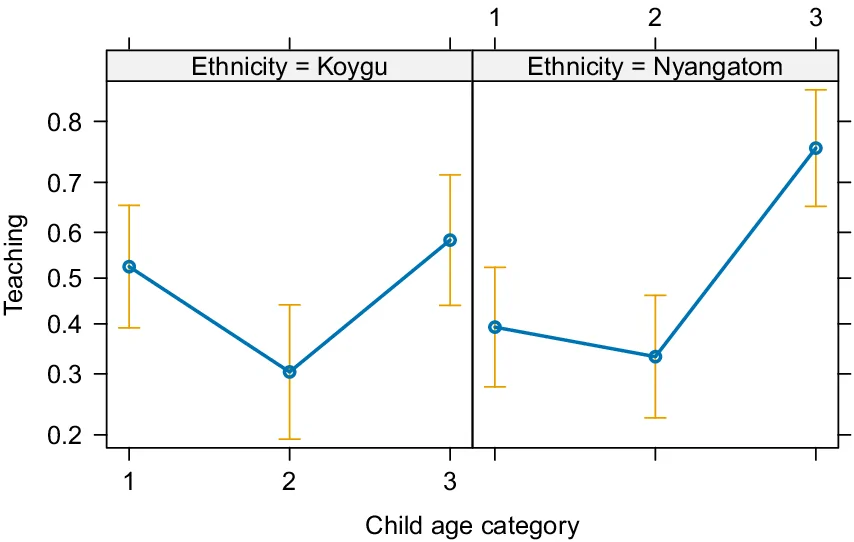
Effects plot of a generalized linear mixed effects model of the relationships between the probability of teaching vs. age, ethnicity, and their interaction, with a random intercept for individual. The model was fit with a binomial distribution and logit link. Error bars are 95% confidence intervals. Age category 1: Early childhood (3–6 years); 2: Middle childhood (7–12 years) and 3: Adolescence (13–18 years). For regression coefficients, see Table 1.

Among Nyangatom, the positive and statistically significant linear age-by-ethnicity interaction indicates a stronger and more consistent increase in teaching with age compared to Koygu. As shown in Figure 4, teaching frequency among Nyangatom is approximately ~40% in early childhood, decreases slightly in middle childhood (~35%), and then increases sharply during adolescence (~75%). However, the quadratic interaction is not statistically significant (*β* = −0.003, *p* = 0.99), suggesting that the overall curvature of the age–teaching relationship is similar across groups. For regression coefficients, see Table 1.

### 3.3 Prediction 3

Given the theoretical and ethnographic evidence that opaque and complex skills and knowledge are acquired later in life (Garfield and Lew-Levy, 2025; Salali et al., 2019), we predict that these features of culture would be more likely to be transmitted in late childhood and adolescence by teaching.

For the purpose of analysis, activities were organized into three categories listed in Table 2: transparent, complex, and opaque. Observed activities were categorized into these three based on a number of criteria. First, during the fieldwork, adult and adolescent participants were shown a list of skills and knowledge from the free-listing analysis (see Bira, 2026 for more details) and were asked to identify which items they found difficult/easy to learn, as well as whether they already knew each skill. This allowed for the identification of skills and knowledge that were difficult to learn (opaque and complex) as well as those that were easy to learn (transparent). Second, the analysis considered whether a skill could be learned through simple observation (transparent) or required some instruction (opaque or complex). Easy to observe and learn skills are called “transparent” activities, abstract cultural knowledge is classified as opaque. Third, the skills were evaluated as to whether they were more abstract or observable (e.g., learning cultural norms versus learning how to herd animals). Fourth, a recent paper by Garfield and Lew-Levy (2025) on teaching and opaque cultural knowledge was reviewed to see if skills and knowledge fell into similar categories. Fifth, in this paper, we use the term opaque culture to refer to causal opacity, as defined by Lyons et al. (2007). Casually opaque tasks are those in which the relationship between an action and its intended goal is not directly perceivable. For further information on the distinctions between instrumental and opaque culture see Garfield and Lew-Levy (2025). These steps helped to identify differences between opaque, complex, and transparent skills or knowledge. Skills that children could learn through observation were classified as transparent. Abstract and socially embedded skills were classified as opaque. Skills or knowledge that were difficult to learn but also observable were classified as complex. See Table 2 for how local skills or knowledge fit into the classification system.

**Table 2:** The three categories of knowledge and skill types based on the criteria developed to test prediction 3.

Transparent	Complex	Opaque
Farming	Complex hair braiding	Music
Cleaning	Process of food preparation	Cultural values/Social norms
Play	Tannery	Knowledge of marriage norms
Fetching water	Making arrow and bow	Gender roles
Toy making	Making boats	Medicinal plant knowledge
Making necklace	Making fire kit	Learning truth finding
Caregiving	Process of making coffee	Knowledge of territorial section
Collecting firewood	Learning to fight	Knowledge of kin terms
Swimming	Building fence/kraals/houses	
Climbing tree	Fishing	
Washing clothes	Learning ecological knowledge	
Cleaning the water bottle	Herding and cattle identification	
Rowing boat	Cattle raiding	
Drawing	Shooting gun	
Milking	Tool making	
Personal safety/Hygiene	Honey production	
	Learning to bargain	
	Sewing clothes	
	Hunting medium and large game	

The results were mixed. We examined the relationship between skill or knowledge type (opaque, complex and transparent) and ethnic group, process of learning (teaching vs. other), and age category (early childhood, middle childhood, and adolescence coded as ordinal values 1, 2, and 3). For the purpose of analysis, the various processes of learning were categorized into two broad groups: teaching and other (see Figure 5). During observations, the following forms of teaching were considered independently: demonstration, feedback, general commands, invitation commands, safety commands, multiple methods of teaching, questioning and answering, stimulus enhancement, storytelling, task assignment, teasing, and verbal instruction. In the analysis, all of these types of teaching were placed into the category of teaching, whereas the remaining, including collaborative learning (role-play and informal play), local enhancement, observation, imitation, practice and pitching-in were placed into the non-teaching category.

**Figure 5:**
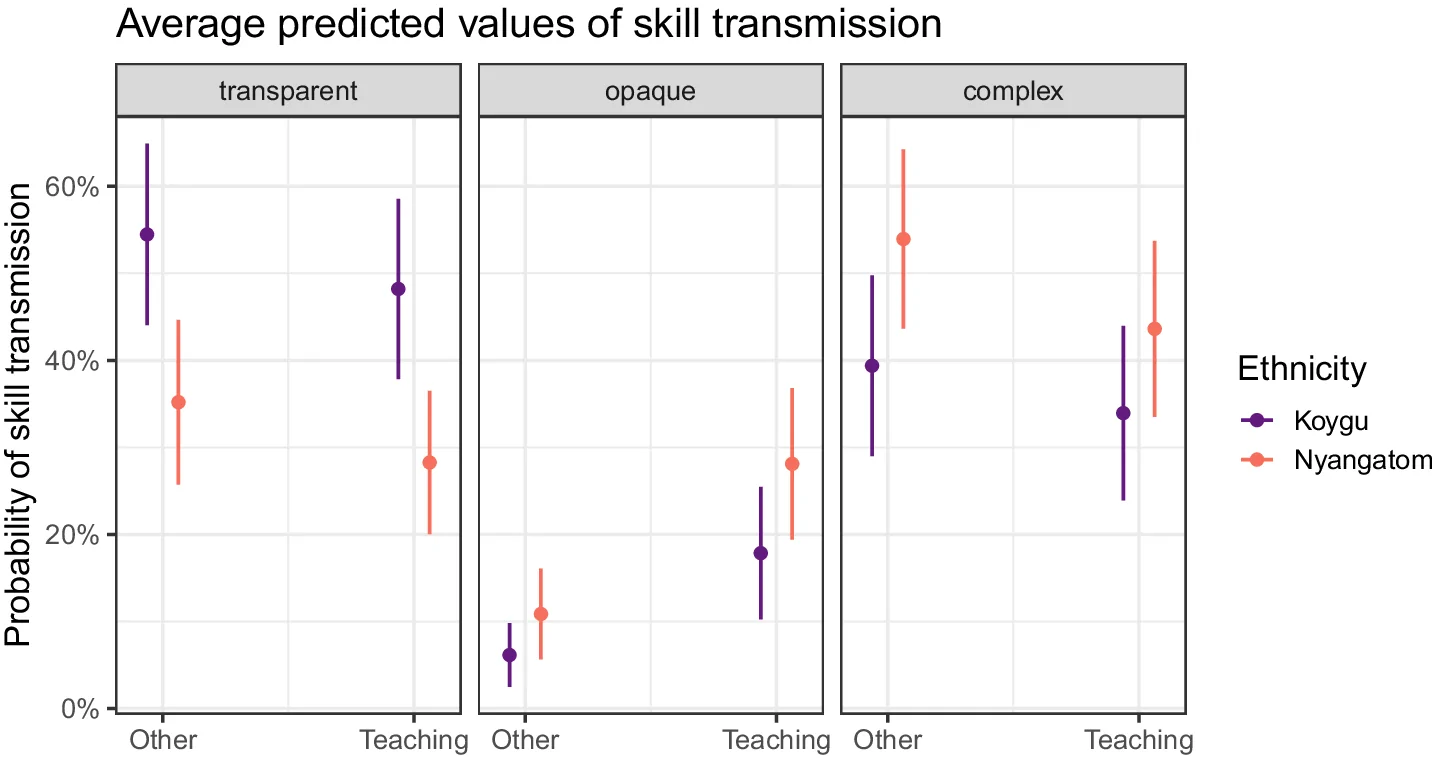
Effects plot of a multinomial logistic mixed effects model of the relationships between the probability of transmitting a transparent, opaque, or complex skill vs. age, ethnicity, and process, with a random intercept for individual. Error bars are 95% confidence intervals. Effects were averaged over the age categories, which were not significantly associated with skill transmission. See Table 1 for regression coefficients and Table 2 for average comparisons.

The results of a multinomial logistic mixed effects regression model found that the ordinal age variable was not significantly associated with nominal skill types, but that there were significant associations with ethnicity and process (Tables 1, 3). Using the marginal effects package (Arel-Bundock et al., 2024), we estimated average comparisons of the nominal skill type outcome with ethnicity and process (Table 3; Figure 5). Koygu were 20% more likely than Nyangatom to engage in transparent skill transmission (*p* = 0.0018), whereas Nyangatom were 7.7% and 12% more likely to engage in opaque and complex skill transmission, respectively, albeit not significantly so (*p* > 0.05). Teaching was significantly positively associated with opaque skill transmission (15%, *p* < 0.001), significantly negatively associated with complex skill transmission (−8.4%, *p* = 0.03), and negatively but not significantly associated with transparent skill transmission (−6.5%, *p* > 0.05). See Table 1 and Figure 5.

**Table 3:** Average comparisons of skill type vs. ethnicity and process from a multinomial logistic mixed effects regression model.

Term	Group	Contrast	Estimate	Std. error	Statistic	*p*-value	Conf. low	Conf. high
Ethnicity	Transparent	Nyangatom—Koygu	−0.200	0.064	−3.10	1.8e-03	−0.320	−0.074
Ethnicity	Opaque	Nyangatom—Koygu	0.077	0.041	1.90	5.7e-02	−0.0022	0.160
Ethnicity	Complex	Nyangatom—Koygu	0.120	0.067	1.80	7.2e-02	−0.011	0.250
Process	Transparent	Teaching—Other	−0.065	0.039	−1.70	9.9e-02	−0.140	0.012
Process	Opaque	Teaching—Other	0.150	0.032	4.60	4.7e-06	0.085	0.210
Process	Complex	Teaching—Other	−0.084	0.039	−2.20	3.0e-02	−0.160	−0.0082

Comparisons estimated with the marginal effects package (Arel-Bundock et al., 2024). See also Table 1 and Figure 5.

Furthermore, child age is not a significant predictor in this model (linear: *β* = −0.36, *p* = 0.24; quadratic: *β* = −0.27, *p* = 0.38), suggesting there is no strong evidence that opaque knowledge and complex skills systematically increase or decrease across ages (See Table 1). Informal observations during a year of fieldwork support the systematic quantitative findings. Several activities described by adults as complex skills were transmitted and acquired by younger children. For example, among the Koygu, a child approximately 4 years old was practicing complex hair-braiding on her mother. This activity requires fine motor coordination, sustained attention, and detailed knowledge of patterning, yet three of the five adults interviewed explicitly described hair braiding as a difficult skill that takes a long time to learn and is often challenging even for adults.

## 4 Discussion

The field study of two cultures with substantially different subsistence activities and foundational schema found both uniformity and diversity in social learning. First, a strong statistical relationship existed between opaque skills or knowledge and teaching. This relationship did not hold for complex skills and knowledge as predicted in previous studies. Second, although the importance of teaching increased with child age, we did not find that opaque or complex cultural skills or knowledge were disproportionately transmitted in middle childhood or adolescence; instead, children began acquiring several of them in early childhood, which is consistent with cross-cultural studies that show children acquire many skills before age seven (Bira and Hewlett, 2023; Garfield et al. 2016). Third, we found that Nyangatom children were most likely to learn from their parents and other adults, while Koygu children were more likely to learn from other children, including siblings. The groups did not differ in the amount of teaching they received from other non-parental adults. In what follows, we discuss these findings in light of ethnographic and evolutionary predictions.

The result that opaque cultural traits were more commonly acquired by various types of teaching than were transparent and complex cultural skills is consistent with previous studies that found teaching is associated with opaque (Garfield and Lew-Levy, 2025) and abstract (Salali et al., 2019) knowledge. The more the cultural traits are abstract or opaque, the more it requires learning by various forms of teaching, including verbal instruction, demonstration and verbal feedback. For instance, among the Nyangatom, children learn context-specific gestures of respect through explicit instruction and immediate feedback when they deviate from expected conduct. Similarly, marriage norms, such as whom one can marry, knowledge about bride price, including the need to be a good herder and cattle raiding, and negotiations around marriage, are learned through storytelling around the fire. The results provide additional evidence to a growing body of research demonstrating that teaching plays an important role in the acquisition of opaque, abstract cultural traits.

Second, the analysis suggested that the importance of teaching increased with child’s age. In relative terms, adolescent children were taught about 1.5 times more than children in early childhood and about 2 times more than children in middle childhood. However, contrary to predictions, opaque and complex cultural traits were not disproportionately transmitted in middle childhood or adolescence. We found that in relative terms, the ratios across age groups were approximately 1, indicating that children in early childhood, middle childhood, and adolescence received learning related to opaque cultural traits and complex skills at nearly the same rate. This finding was inconsistent with previous studies (see Garfield and Lew-Levy, 2025; Kline et al., 2013; Schniter et al., 2015) that found abstract knowledge, difficult tasks or opaque culture are acquired from middle childhood onwards.

A possible explanation for this difference is partly related to the methodology. For instance, our study only focused on children from 3 to 18 years old whereas Kline et al. (2013) and Schniter et al. (2015) study included adults. Furthermore, it is important to note that the timing of the acquisition of opaque and complex cultural traits is impacted by factors other than age. As Rogoff (2003) argues, child development is a cultural process in which learning of cultural traits occurs through participation in everyday life. For instance, a young child of 4 years old was observed practicing complex hair braiding in Koygu, skills that three of the five adult women mentioned as difficult to learn. Additionally, honey production is among the complex skills involving multiple forms of knowledge to make a beehive, including identifying good trees to place to hang it, and how to collect the honey from the beehives, which means teaching could have been received from several individuals over time. In the riverine forest next to the Omo River, many male members engage in making beehives; some of them hang a beehive, and some of them bring honey from the beehive. Learning such complex skills is a cumulative process that children are first exposed to in early childhood, even before knowing what it is. Then, in late childhood or adolescence, their father or elder brother takes them with them to the place to make a beehive, hangs a beehive, or collect honey, and explicitly tells them or demonstrates to them so that they can make it themselves another time. Thus, in small-scale cultures, children participate in everyday activities from early childhood, exposing them to complex skills that require a certain degree of specialization well before middle childhood. The results were instead consistent with Lew-Levy et al. (2021) study among the Hadza of Tanzania and BaYaka of the Republic of Congo, which found that task difficulty/complexity was not strongly associated with age of acquisition.

Lastly, we found that Nyangatom children learned primarily from their parents or other adults, while Koygu children were more likely to learn from other children, including siblings, and the groups did not differ in the amount of teaching received from other non-parental adults. The evolutionary concept of cultural niche construction (Laland et al., 2001) helps explain the cross-cultural variation in terms of who teaches children. Previous studies among Aka hunter-gatherers and Ngandu farmers found that foundational schema is part of these niches to which children have to adapt and they shape social learning in children (Boyette and Hewlett, 2017). When we compare the Nyangatom and the Koygu, the Nyangatom pastoralists are characterized by a hierarchical foundational schema (Bedasso, 2016; Bira, 2026; Tornay, 1981), whereas the Koygu have an egalitarian foundational schema similar to other hunter-gatherers. The hierarchical nature of the Nyangatom foundational schema is based on the principle of respect for hierarchy, especially respect for elders, which in turn facilitates hierarchical teaching by older individuals (i.e., by parents) through task assignment, verbal feedback and different forms of commands. In contrast, the egalitarian foundational schema of the Koygu provides children greater autonomy to navigate their environments by themselves. Constraints do not exist for children teaching other children and adults minimize their interventions in children’s learning, thereby facilitating horizontal teaching among children of the same generation.

It is important to recognize the agency of children in this process as well. Children move through the social environment and learn from others. Others are not always deciding when and what to transmit to a child. Children move about and engage in activities with people around them and learning events occur. Results indicate that pastoralist transmission is primarily vertical, but this is in part due to children being near or with parents and observing their skills or knowledge. The mode is coded as “vertical transmission” which may give the impression that the parent initiates the learning event but instead it starts by the child interests’ in learning the skill by observing, imitating or practicing what a parent is doing. The child is motivated on his or her own to observe their mother grinding or their father herding a goat in part because of the parent’s proximity to the child. The parent does not say “watch me” because this is important to learn.

Several studies have suggested that teaching is a mechanism for the transmission of opaque or difficult or cumulative culture (Boyette and Hewlett, 2017; Garfield and Lew-Levy, 2025; Lew-Levy et al., 2018; Salali et al., 2019). As cultural traits become more complex, cumulative cultural transmission requires a high-fidelity transmission to ensure accurate transmission from one generation to the next or from group to another within a generation (Fogarty et al., 2011). Teaching was among the high-fidelity transmission and culturally adaptive mechanisms for transmitting abstract and complex cultural traits (Garfield and Lew-Levy, 2025; Salali et al., 2019). Opaque cultural traits present a well-recognized problem for social learning. The results suggest that teaching mitigates this issue by making opaque or abstract cultural knowledge accessible to learners through storytelling, verbal explanation, demonstration, feedback, or pedagogical questioning and answering. By providing explanations, demonstrations with intentional emphasis, and corrective feedback, teachers reduce inferential load and increase transmission accuracy, thereby enabling the persistence of opaque cultural traits. The association between teaching and opaque cultural traits is statistically significant, but complex skills are not. This suggests that future studies that involve learning of complex skills can look into how teaching and other processes of learning jointly contribute into the transmission of complex cultural behaviors.

The results generally support cultural evolutionary approaches that conceptualize teaching as a relatively unique and critical adaptation for understanding human cultural evolution. Teaching does not replace other social learning strategies, such as imitation or emulation. Instead, it operates with them and becomes particularly important when information is opaque, or abstract. By enhancing fidelity under these conditions, teaching supports the accumulation and persistence of opaque cultural traditions across generations.

## 5 Limitations of the study

First, although our sample size is consistent with previous focal follow methodologies, it may constrain our ability to detect higher-order interactions (e.g., between ethnicity, gender, and task type). This limitation is particularly relevant for interpreting null or marginal effects, which may reflect limited statistical power rather than true absence of associations.

Second, the presence of an observer or a researcher can sometimes trigger “pedagogical performances” by parents or guardian. Although it was impossible to totally overcome this situation, our observation shows that in most observed cases of vertical and oblique transmission, learning interactions were initiated by the children themselves rather than prompted by adults. Parents and other adults typically responded to children’s ongoing activities by providing assistance or guidance, rather than staging instructional behavior.

## 6 Conclusion

This study examined how children in two culturally distinct communities acquired complex cultural skills and knowledge. Theories and proposals from previous studies were tested and found that (1) foundational schema impact social learning, with more vertical or oblique learning from older individuals among Nyangatom pastoralists and more horizontal peer learning among Koygu foragers; (2) teaching increases with age, with adolescents receiving substantially more teaching than younger children; and (3) teaching was associated with the transmission of opaque culture. Although complex skills were largely acquired by teaching, the difference between teaching and other types of process of transmission were statistically not significant. Furthermore, opaque and complex cultural traits were not disproportionately transmitted in middle childhood or adolescence.

## Data Availability

Publicly available datasets were analyzed in this study. This data can be found here: https://github.com/grasshoppermouse/ethiopia2024.
